# The diagnostic dilemma: Giant Mucocele of the appendix—challenging conventional thinking

**DOI:** 10.1093/jscr/rjad171

**Published:** 2023-04-12

**Authors:** Paola Solis-Pazmino, Cynthia Cedillo, Augusta Avila, Luis Henrique Saldanha, Vitor Doncatto, Mohamed Hamaoui

**Affiliations:** Surgery Department, Santa Casa de Misericórdia in Porto Alegre (SCMPA), Porto Alegre, Brazil; Surgery Department, Santa Casa de Misericórdia in Porto Alegre (SCMPA), Porto Alegre, Brazil; Surgery Department, Santa Casa de Misericórdia in Porto Alegre (SCMPA), Porto Alegre, Brazil; Surgery Department, Santa Casa de Misericórdia in Porto Alegre (SCMPA), Porto Alegre, Brazil; Surgery Department, Santa Casa de Misericórdia in Porto Alegre (SCMPA), Porto Alegre, Brazil; Surgery Department, Santa Casa de Misericórdia in Porto Alegre (SCMPA), Porto Alegre, Brazil

**Keywords:** appendix, mucocele, neoplasm, appendicitis, ileocolectomy

## Abstract

This article reports on a 76-year-old male patient diagnosed with a mucocele of the appendix. A mucocele of the appendix is a benign fluid-filled swelling caused by the accumulation of mucus in the appendix due to a blockage in its discharge route. The patient presented with symptoms of chronic constipation and rectal tenesmus and was diagnosed through physical examination, imaging studies and laboratory tests. The patient underwent an open ileocolectomy procedure to remove the affected appendix. The results of the histopathological analysis showed a confined low-grade appendiceal mucinous neoplasm. The overall survival rate after surgery for mucocele of the appendix is excellent, with a low recurrence rate.

## INTRODUCTION

A mucocele of the appendix is a benign, fluid-filled swelling in the appendix, a narrow pouch that projects from the large intestine. It is caused by the accumulation of mucus in the appendix due to a blockage in its normal discharge route. The incidence ranges from 0.1 to 0.7% post-appendectomy [1].

This condition can result in the enlargement of the appendix, leading to pain and discomfort [2]. If left untreated, a mucocele of the appendix can lead to complications, including infection and appendix rupture. Treatment typically involves surgical removal of the affected appendix [4].

Accurately classifying appendiceal mucinous lesions is crucial for determining the appropriate treatment and prognosis. Histology categorizes these lesions into two groups: non-neoplastic, such as retention cysts or inflammatory mucoceles, and neoplastic, including serrated lesions, hyperplastic polyps, low-grade appendiceal mucinous neoplasms (LAMNs), high-grade appendiceal mucinous neoplasms (HAMNs) and mucinous adenocarcinomas. Understanding the type of lesion is essential for making informed medical decisions and ensuring the best outcomes for patients [3]. This article aims to report a 76-year-old male patient with the diagnostic and management of mucocele.

## CASE REPORT

A 76-year-old man with a history of hypertension was admitted to the digestive surgery service after experiencing two years of chronic constipation and rectal tenesmus. Despite these symptoms, the patient reported no abdominal pain, rectal bleeding or weight loss.

Upon physical examination, the patient reported mild tenderness in the right lower quadrant upon palpation, but there was no indication of peritonism. The digital rectal examination revealed a normal anal tone and an empty rectum with no evidence of bleeding. Additionally, the cardiorespiratory examination showed no abnormal findings.

**Figure 1:**
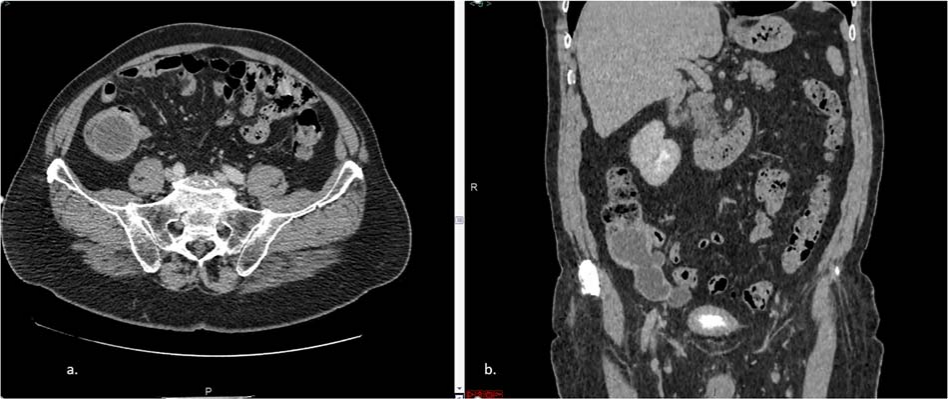
**a**. Axial CT section of the abdomen showing signs of mucocele of the appendix with intraluminal air. **b**. The coronal CT section of the appendix shows that the anterior wall of the appendix had an irregular thickening close to the transition of the middle/distal third.

The abdominal CT scan showed cystic distension of the cecal appendix with a maximum diameter of 3.6 cm, accompanied by intussusception into the cecum. The anterior wall of the appendix showed irregular thickening close to the transition of the middle/distal third (Fig. 1). The colonoscopy revealed a mount-like elevation of the appendix orifice, also known as the ‘volcano sign’. The patient’s laboratory test results showed a mild elevation of the cancer-associated antigen 19–9 (52 U/ml), with normal levels of CEA and white blood cells (WBC). The patient’s renal function and electrolyte levels were within normal limits.

## TREATMENT

**Figure 2:**
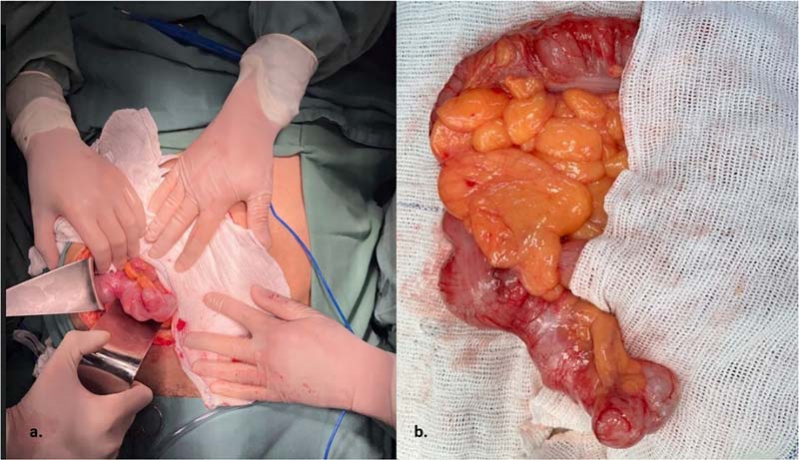
The intraoperative revealed a cystic mass measuring 9.2 × 3.4 × 3.0 cm was found in the right iliac fossa.

The patient underwent an elective surgical procedure. During the operation, a cystic mass measuring 9.2 × 3.4 × 3.0 cm was found in the right iliac fossa (Fig. 2). The surgical team released Told’s line and the proximal transverse and ascending colon until reaching the terminal ileum and resected the mass with a safety margin. An ileocolectomy with a primary anastomosis was performed using a 75-mm linear stapler reinforced with a continuous suture for hemostasis. This surgical intervention effectively addressed the cystic mass in the patient’s abdomen.

**Figure 3:**
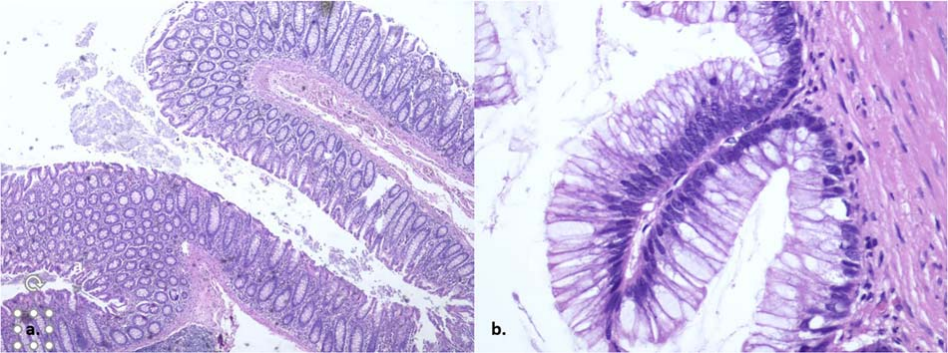
The histopathology showed a low-grade appendiceal mucinous neoplasm with no rupture and clear margins.

The pathology report unveils a confined low-grade appendiceal mucinous neoplasm with no rupture and clear margins (Fig. 3). The patient achieves a successful outcome with consistent monitoring and no complications following surgery.

## DIFFERENTIAL DIAGNOSIS

Mucocele of the appendix is a condition with symptoms that can mimic other illnesses. Thus, it is crucial to perform a differential diagnosis to eliminate other possible causes of the patient’s symptoms. Possible differential diagnoses for mucocele of the appendix include appendicitis, ovarian cyst, diverticulitis, Crohn’s disease and gastrointestinal tumors. A comprehensive evaluation that incorporates the patient’s medical history, physical examination, imaging studies and laboratory tests may be required to make an accurate diagnosis. Having a precise diagnosis is essential to achieve optimal treatment outcomes and minimize the likelihood of complications.

## DISCUSSION

Appendiceal mucocele is a term that refers to the accumulation of mucin in the appendix, as seen in imaging studies [5]. However, the true nature and behavior of the condition can only be determined through histopathological analysis [6]. In this case report, we present the case of an older adult who was unexpectedly diagnosed with mucocele of the appendix.

Due to the unspecific symptoms that can range from non to acute pain in the right lower quadrant, a preoperative clinical diagnosis of mucocele can be challenging [7]. However, an initial suspicion of the condition is essential to avoid complications during surgery, such as pseudomyxoma peritoneal [1].

The optimal surgical approach for the mucocele of the appendix remains controversial, with some studies supporting laparoscopic surgery as safe and feasible. In contrast, others recommend open surgery based on clinical experience [8]. In this case, the patient underwent an open ileocolectomy procedure as the appendix base was involved in the disease process. In a systematic review of 276 patients with non-perforated AMs, just 13% perform ileocolectomy [9].

Histology is crucial in determining the treatment and prognosis of appendiceal mucinous lesions [10]. The results of the histopathological analysis, in this case, showed a confined low-grade appendiceal mucinous neoplasm without rupture and with negative margins, which meant that no additional treatment was required.

The overall survival rate after initial surgery for mucocele of the appendix is excellent, with a low recurrence rate of 3–7% for patients with acellular mucin deposits but a higher risk of recurrence of 33–78% with cellular mucin outside of the appendix [3].

## CONCLUSION

The mucocele of the appendix is a noncancerous buildup of mucus caused by a blockage. Proper diagnosis is essential for treatment and prognosis and may involve various medical evaluations. The best surgical approach is debated, but histology is crucial for treatment decisions. The survival rate after surgery is high, but the recurrence rate varies based on the presence of cellular mucin.

## CONFLICT OF INTEREST STATEMENT

The authors declare that there is no conflict of interest.

## FUNDING

The authors declare that there is no funding support for this manuscript.

## AUTHORS' CONTRIBUTIONS

Paola Solis- Pazmino and Mohamed Hamaoui: conceived and designed the work. Paola Solis-Pazmino, Cynthia Cedillo and Augusta Avila drafted the manuscript. Luis Henrique Saldanha, Vitor Doncatto: data collection. Paola Solis- Pazmino and Mohamed Hamaoui: corrections and final article review.

All authors read and approved the final manuscript version to be published.

## ETHICAL STANDARDS

All procedures followed were by the ethical standards of the responsible committee on human experimentation (institutional and national) and with the Helsinki Declaration of 1975, as revised in 2008.

## INFORMED CONSENT

Informed consent was obtained from the patient to be included in the study.
